# Chronic cough caused by cerebellar tonsillar herniation: a case report

**DOI:** 10.1186/s12890-026-04504-8

**Published:** 2026-07-18

**Authors:** Na Li, Jiangying Guo, Yue Hu, Zihan Yi, Guangyu Ying, Huaqiong Huang

**Affiliations:** 1 Key Laboratory of Respiratory Disease of Zhejiang Province, Department of Respiratory and Critical Care Medicine, Second Affiliated Hospital of Zhejiang University School of Medicine, Hangzhou, Zhejiang Province 310009 China; 2 Department of Neurosurgery, The Second Affiliated Hospital of Zhejiang University School of Medicine, Hangzhou, Zhejiang Province 310009 China

**Keywords:** Chronic cough, Cerebellar tonsillar herniation, Water swallow test, MDT clinic, Case report

## Abstract

**Background:**

Chronic cough is most commonly associated with respiratory or gastroesophageal disorders, whereas central nervous system etiologies are rare. Cerebellar tonsillar herniation is typically characterized by neurological manifestations, but cough as an isolated symptom is uncommon.

**Case presentation:**

A 61-year-old woman presented with a one-year history of recurrent cough that worsened after eating or drinking. Initial respiratory, gastrointestinal, and allergic evaluations, including chest computed tomography (CT), laboratory testing, and bronchial challenge, were unremarkable. Multidisciplinary team assessment prompted a water swallow test, which showed coughing episodes, raising suspicion for central nervous system involvement. Subsequent magnetic resonance imaging revealed cerebellar tonsillar herniation with 10-mm descent below the foramen magnum. The patient underwent posterior fossa decompression with occipital-cervical fusion. Her cough resolved completely postoperatively, with sustained symptom remission at one-year follow-up.

**Conclusions:**

Cerebellar tonsillar herniation may present solely with chronic cough, particularly when common etiologies have been excluded. Thorough clinical assessment, including simple bedside tools such as the water swallow test, and multidisciplinary evaluation can promote timely diagnosis. Cerebellar tonsillar herniation should be regarded as a potential etiologic factor in the differential diagnosis of chronic cough.

## Background

Chronic cough is defined as a persistent cough lasting more than 8 weeks, with no significant findings on chest X-ray. The majority of chronic cough cases are attributed to conditions, such as cough variant asthma (CVA), gastroesophageal reflux-induced cough (GERC), eosinophilic bronchitis (EB), upper airway cough syndrome (UACS), and allergic cough (AC), collectively accounting for over 70% of cases [1]. Rare causes of chronic cough involve a wide range of conditions, including disorders of the throat, trachea, lungs, mediastinum, and cardiovascular system [2]. A particularly rare case of chronic cough caused by cerebellar tonsillar herniation was presented in this study.

## Case presentation

A 61-year-old woman presented with a one-year history of recurrent cough. The cough was initially paroxysmal and of unexplained origin, accompanied by the expectoration of white sputum. During the antecedent 12-month interval, the patient manifested a chronic, daily tussive syndrome characterized by a frequency ranging from several to in excess of ten episodes per diem, with individual symptomatic events exhibiting durations lasting from seconds to tens of seconds. The symptoms worsened after eating or drinking. The patient denied associated symptoms, such as chest tightness, shortness of breath, dizziness, syncope, acid reflux, hiccups, nasal congestion, rhinorrhea, hoarseness, limb numbness, or fever. Chest computed tomography (CT) scan conducted one month ago revealed no significant abnormalities. Despite treatment with various cough medications, including dextromethorphan, the patient did not experience significant improvement and subsequently sought further evaluation in the respiratory department. The patient also denied a history of allergies, smoking, or exposure to dust and harmful gases. On physical examination, bilateral breath sounds were normal, and chest expansion was symmetric, and no positive signs were identified. Laboratory tests, including white blood cell count, eosinophil count, serum immunoglobulin E, liver and kidney function tests, electrolytes, electrocardiogram, and cardiac ultrasound, were all within normal limits. The bronchial challenge test was negative. Given the absence of a clear etiology for the patient’s cough based on the history and examination, referral to a multidisciplinary team (MDT) clinic, including a respiratory specialist, gastroenterologist, and otolaryngologist, was recommended for further evaluation of chronic cough [3]. The patient underwent a more detailed examination. A more concentrated clinical history revealed that the patient’s tussive episodes were characteristically exacerbated by the initiation of oral intake. Crucially, this symptomatic worsening occurred in the absence of concurrent gastrointestinal manifestations, such as nausea, emesis, acid reflux, or singultus. Consequently, a standardized water swallow test was indicated to evaluate for potential oropharyngeal dysphagia [4]. The patient sat upright and drank 30 mL of warm boiled water, which she was able to swallow in one attempt, although symptoms of coughing were noted.

**Fig. 1:**
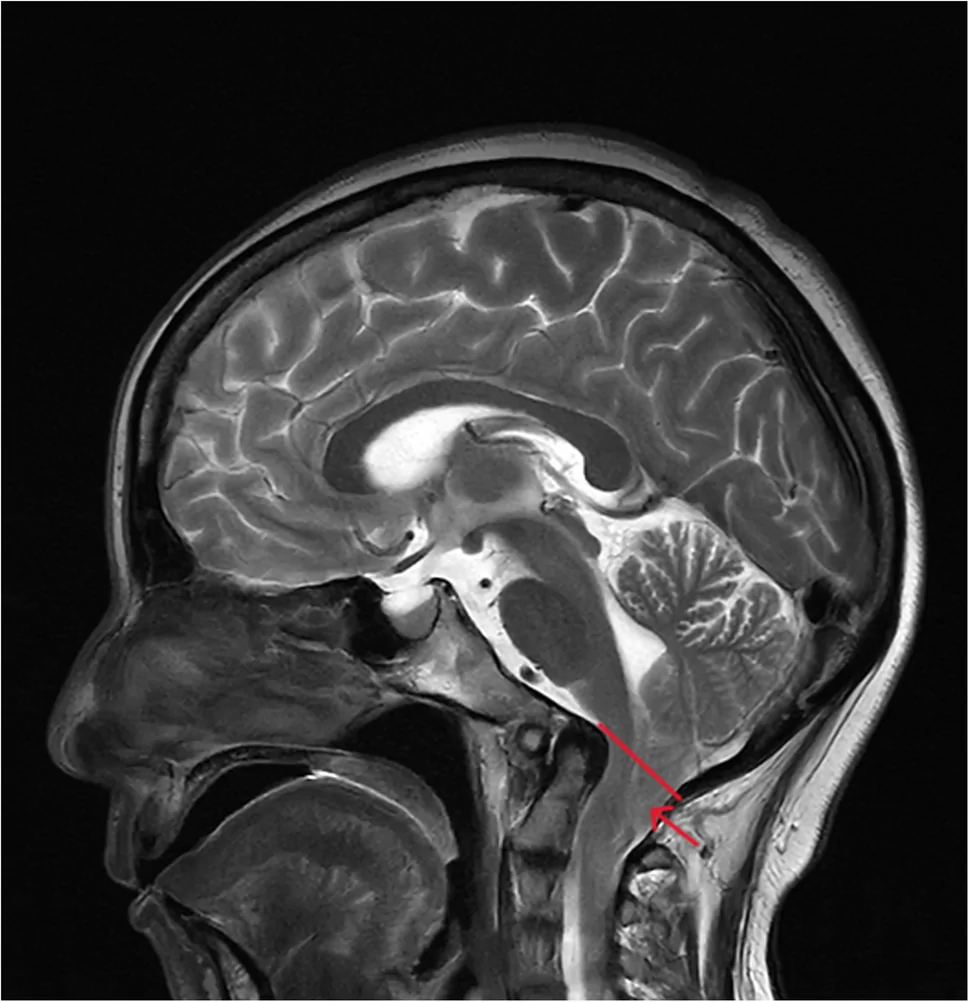
Cerebellar tonsillar ectopia (arrow): the inferior margin of the tonsils is located 10 mm below McRae’s line (the red reference line)

**Fig. 2:**
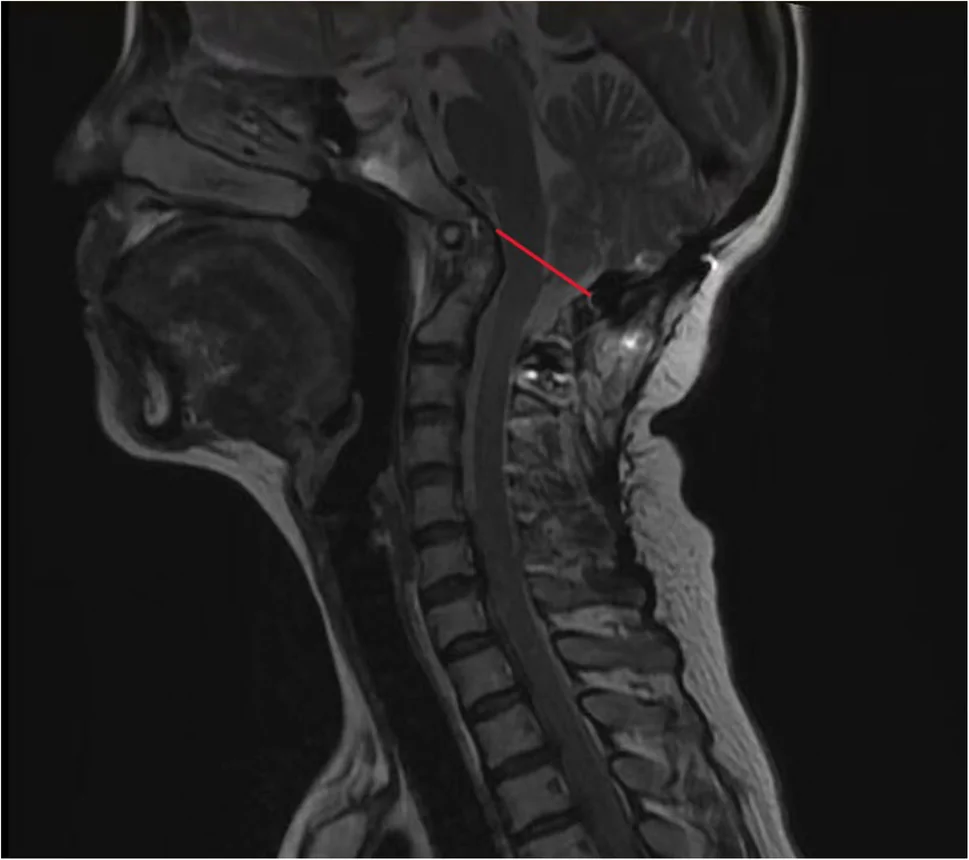
Post-surgical sagittal MRI of the brain shows the inferior margin of the cerebellar tonsils not extending beyond McRae’s line

The positive result of the water swallow test confirmed the presence of deglutitive dysfunction. Following a comprehensive clinical evaluation and MDT deliberation, a multi-system diagnostic strategy was conceptualized to systematically investigate potential central nervous system (CNS), laryngological, and gastroesophageal etiologies; this planned diagnostic workup included cranial magnetic resonance imaging (MRI), flexible laryngoscopy, and gastroscopy. To evaluate suspected neurogenic pathology, cranial MRI was performed, revealing basilar invagination with skull base depression and associated cerebellar tonsillar herniation, notably in the absence of syringomyelia. Quantitatively, the cerebellar tonsils demonstrated a caudal descent of 10 mm below the McRae line, indicating the anatomical plane of the foramen magnum (Fig. 1). Upon neurosurgical consultation, surgical intervention was recommended, and the patient was subsequently transferred to the neurosurgery department for specialized management. A concentrated, high-resolution neurological examination revealed mildly diminished strength in the neck extensor musculature, generalized tendon hyperreflexia (+++) across all four limbs, and a positive Hoffmann’s sign. These objective upper motor neuron signs strongly validated a CNS origin for the patient’s atypical tussive reflex, thereby indicating that further invasive diagnostic procedures, such as laryngoscopy and gastroscopy, are clinically redundant. She subsequently underwent for posterior foramen magnum decompression, combined with occipital-cervical fusion and internal fixation. The surgery was successful, and the patient’s cough significantly improved postoperatively. Two months after the surgery, the patient returned for a follow-up visit. The patient had no obvious cough symptoms, and MRI showed no cerebellar tonsillar herniation (Fig. 2). One year after the surgery, the patient returned for follow-up and still had no obvious cough symptoms.

### Discussion and conclusions

Most cases of chronic cough are attributed to conditions, such as CVA, GERC, EB, UACS, and AC. However, approximately 7% of patients with chronic cough have no identifiable cause [1, 5, 6]. Through the collaborative efforts of a MDT, medical history and examination can be approached from different perspectives, promoting a quicker identification of the underlying cause and enabling timely treatment [3]. It was due to the patient’s referral to the MDT clinic that the cause of the cough was promptly identified. Therefore, MDT involvement is crucial in the diagnosis and management of refractory chronic cough, playing a significant role in its effective treatment.

Cerebellar tonsillar herniation is a congenital malformation of the posterior fossa, characterized by the herniation of the lower part of the cerebellar tonsils below the level of the foramen magnum. This condition can lead to a variety of clinical symptoms, resulting from compression of the herniated cerebellar tonsils on the medulla, posterior cranial nerves, or interference with cerebrospinal fluid circulation. Common symptoms include occipital neck pain, facial paresthesias, ataxia, nystagmus, dysphagia, water choking, hoarseness, etc. The diagnostic criterion for cerebellar tonsillar herniation is an extension of the tonsils more than 5 mm below the foramen magnum [7–9]. In this case, the patient was diagnosed with cerebellar tonsillar herniation, presenting with cough as the sole symptom and without the usual neurological signs associated with the condition. This atypical presentation delayed the diagnosis for over a year. In the present case, the patient’s cough was ultimately attributable to cerebellar tonsillar herniation. The pathogenic mechanism relevant to our clinical observation is the compression of the medulla oblongata and the lower cranial nerves (particularly the glossopharyngeal and vagus nerves) by the ectopic tonsils. This compression disrupts the bulbar swallowing center and impairs the efferent motor output to the pharyngeal constrictors and laryngeal muscles, resulting in inadequate glottic closure and diminished airway protection during liquid ingestion. Consequently, the premature extravasation of the pharyngeal bolus into the laryngeal vestibule and subsequent trachea elicits the cough reflex, a vital physiological defense mechanism which, in this clinical population, paradoxically manifests as a pathognomonic sentinel sign indicative of an underlying structural anomaly involving the central neuroanatomical control centers of the brainstem [10]. The patient’s cough was resolved postoperatively that could be attributed to the relief of compression on the medulla oblongata and lower cranial nerves.

Moreover, cerebellar tonsillar herniation is a congenital anomaly and represents a very rare cause of chronic cough, particularly in children, with minimal reported cases in young and middle-aged adults, and no previous reports in elderly patients [11–14]. Upon further detailed history taking, it was noted that the patient’s cough worsened after drinking water and eating. A simple and feasible water swallow test was performed, yielding a positive result. The water swallow test is a simple and effective method for assessing a patient’s swallowing function, revealing their behavior and performance while drinking water [15]. A positive water swallow test provides a rapid indication of swallowing dysfunction. The differential diagnosis of dysphagia is broad and includes neurogenic disorders, oropharyngeal and laryngeal pathology, and age-related degenerative changes, etc. Accordingly, clarification of the underlying cause requires careful integration of the patient’s medical history, physical examination, and targeted ancillary investigations. In the present case, the MDT developed a stepwise diagnostic plan. Because the patient was an elderly woman and MRI is a noninvasive modality, MRI was performed at an early stage. The imaging findings of cerebellar tonsillar herniation prompted neurosurgical consultation, and subsequent neurological examination revealed additional abnormal signs. On this basis, further laryngoscopic or gastroscopic evaluation was not pursued, and the patient ultimately underwent surgical treatment. Postoperatively, the cough resolved completely and did not recur during the one-year follow-up. Nevertheless, the absence of videofluoroscopic swallowing study (VFSS) and fiberoptic endoscopic evaluation of swallowing (FEES) represents a limitation of this case report. Inclusion of these objective swallowing assessments, together with gastroscopy to further evaluate gastrointestinal causes such as reflux and laryngoscopy to exclude local laryngeal pathology, would have strengthened diagnostic differentiation and provided more comprehensive evidence to support the clinical management of this patient.

In conclusion, this is the first reported case of an elderly patient with cerebellar tonsillar herniation presenting with chronic cough, a condition that is extremely rare. In patients with refractory chronic cough, it is essential to not only screen for common etiologies, but also to conduct a thorough history and physical examination to identify less common causes, such as CNS-related disorders, including cerebellar tonsillar herniation.

## Data Availability

All data supporting the findings of this study are included in this published article. Further enquiries can be directed to the corresponding author.

## Abbreviations

CVA: Cough variant asthma
GERC: Gastroesophageal reflux-induced cough
EB: Eosinophilic bronchitis
UACS: Upper airway cough syndrome
AC: Allergic cough
CT: Computed tomography
MDT: Multidisciplinary team
MRI: Magnetic resonance imaging
